# Tomato histone H2B monoubiquitination enzymes SlHUB1 and SlHUB2 contribute to disease resistance against *Botrytis cinerea* through modulating the balance between SA- and JA/ET-mediated signaling pathways

**DOI:** 10.1186/s12870-015-0614-2

**Published:** 2015-10-21

**Authors:** Yafen Zhang, Dayong Li, Huijuan Zhang, Yongbo Hong, Lei Huang, Shixia Liu, Xiaohui Li, Zhigang Ouyang, Fengming Song

**Affiliations:** National Key Laboratory for Rice Biology, Institute of Biotechnology, Zhejiang University, Hangzhou, 310058 China

**Keywords:** *Botrytis cinerea*, Defense response, Histone H2B ubiquitination, RING E3 ligase, Signaling pathway, Tomato (*Solanum lycopersicum*)

## Abstract

**Background:**

Histone H2B monoubiquitination pathway has been shown to play critical roles in regulating growth/development and stress response in Arabidopsis. In the present study, we explored the involvement of the tomato histone H2B monoubiquitination pathway in defense response against *Botrytis cinerea* by functional analysis of SlHUB1 and SlHUB2, orthologues of the Arabidopsis AtHUB1/AtHUB2.

**Methods:**

We used the TRV-based gene silencing system to knockdown the expression levels of *SlHUB1* or *SlHUB2* in tomato plants and compared the phenotype between the silenced and the control plants after infection with *B. cinerea *and *Pseudomonas syringae* pv. *tomato (Pst)* DC3000. Biochemical and interaction properties of proteins were examined using *in vitro* histone monoubiquitination and yeast two-hybrid assays, respectively. The transcript levels of genes were analyzed by quantitative real time PCR (qRT-PCR).

**Results:**

The tomato SlHUB1 and SlHUB2 had H2B monoubiquitination E3 ligases activity *in vitro* and expression of *SlHUB1* and *SlHUB2* was induced by infection of *B. cinerea* and *Pst* DC3000 and by treatment with salicylic acid (SA) and 1-amino cyclopropane-1-carboxylic acid (ACC). Silencing of either *SlHUB1* or *SlHUB2* in tomato plants showed increased susceptibility to *B. cinerea*, whereas silencing of *SlHUB1* resulted in increased resistance against *Pst* DC3000. SlMED21, a Mediator complex subunit, interacted with SlHUB1 but silencing of *SlMED21* did not affect the disease resistance to *B. cinerea* and *Pst* DC3000. The *SlHUB1*- and *SlHUB2*-silenced plants had thinner cell wall but increased accumulation of reactive oxygen species (ROS), increased callose deposition and exhibited altered expression of the genes involved in phenylpropanoid pathway and in ROS generation and scavenging system. Expression of genes in the SA-mediated signaling pathway was significantly upregulated, whereas expression of genes in the jasmonic acid (JA)/ethylene (ET)-mediated signaling pathway were markedly decreased in *SlHUB1*- and *SlHUB2*-silenced plants after infection of *B. cinerea*.

**Conclusion:**

VIGS-based functional analyses demonstrate that both SlHUB1 and SlHUB2 contribute to resistance against *B. cinerea* most likely through modulating the balance between the SA- and JA/ET-mediated signaling pathways.

**Electronic supplementary material:**

The online version of this article (doi:10.1186/s12870-015-0614-2) contains supplementary material, which is available to authorized users.

## Background

To defend attack from potential pathogens, plants have evolved to possess multilayer of immune responses [1]. The first layer is triggered upon the detection of pathogen- or microbial-associated molecular patterns (PAMPs/MAMPs) by pattern recognition receptors on the external face of plant cells and is called PAMP-triggered immunity (PTI) [2]. To circumvent PTI, pathogens evolve to produce a large number of effectors, which are delivered into plant cells to suppress PTI and facilitate pathogenesis [2, 3]. As a counter measure, plants have acquired additional intracellular receptors called resistance (R) proteins to recognize pathogen effectors, resulting in initiation of the second layer of defense, known as effector-triggered immunity (ETI) [1, 4–6]. Generally, ETI is quantitatively stronger and longer-lasting than PTI; however, initiation of both PTI and ETI often requires expression reprogramming of a plenty of genes [7–10]. Recently, extensive genetic and biochemical studies have shown that ubiquitin-mediated protein modification plays critical roles in plant immune responses [8, 11–15].

Ubiquitin-mediated protein modification has been demonstrated to play critical roles in regulation of growth, development, senescence [16–18], abiotic stress responses [19], hormone signaling [20–22], and immune responses against pathogens [23–25]. Ubiquitination can be classified into two major types, namely monoubiquitination and polyubiquitination, depending on whether a single ubiquitin moiety or a polymerized ubiquitin chain is attached to target proteins [24]. Polyubiquitination generally leads to the degradation of the target proteins through the 26S proteasome [26] while monoubiquitination of target proteins does not lead to degradation by the proteasome. Instead, monoubiquitination functions as an endogenous signal [27].

Histone monoubiquitination together with other types of posttranslational modifications can modulate nucleosome/chromatin structure and DNA accessibility and thus regulate diverse DNA-dependent processes [28–32]. Monoubiquitinated histone H2B (H2Bub1) was detected widely throughout eukaryotes spanning from yeast to humans and plants [29, 30, 33, 34]. In Arabidopsis, H2Bub1 is associated with active genes distributed throughout the genome and marks chromatin regions notably in combination with histone H3 trimethylated on K4 (H3K4me3) and/or with H3K36me3 [35]. During early photomorphogenesis, gene upregulation was found to be associated with H2Bub1 enrichment [36]. Recent studies have suggested the involvement of HISTONE MONOUBIQUITINATION1 (AtHUB1)- and AtHUB2-mediated histone H2B monoubiquitination in Arabidopsis growth and development. It has been demonstrated that AtHUB1 and AtHUB2 act nonredundantly in the same pathway and play important roles in regulation of early leaf and root growth [37], cuticle composition [38], seed dormancy [39], vegetative and reproductive development [40], photomorphogenesis [36, 41], flowering and floral transition [42–44].

It was recently demonstrated that the histone H2B monoubiquitination acts as an important type of chromatin modifications with regulatory roles in plant immune responses. The Arabidopsis *athub1* mutant plants showed increased susceptibility to *Botrytis cinerea* and *Alternaria brassicicola*, two typical necrotrophic fungal pathogens, but did not alter the response to *Pseudomonas syringae* pv. *tomato* (*Pst*) DC3000 [13]. Both of AtHUB1 and AtHUB2 mediated histone H2B monoubiquitination directly at *SNC1*, the *SUPPRESSOR OF npr1-1, CONSTITUTIVE1* gene, and loss of *AtHUB1* or *AtHUB2* function reduced the upregulation of *SNC1* expression and suppressed the *bon1* autoimmune phenotypes [45]. It was found that the function of *AtHUB1* was independent on jasmonate, but ethylene (ET) responses and salicylic acid (SA) was involved in the resistance of *athub1* mutants to necrotrophic fungi [13]. Furthermore, AtHUB1 interacted with AtMED21, a subunit of the Arabidopsis Mediator complex, and RNAi-mediated supression of *AtMED21* expression also led to increased susceptibility to *B. cinerea* and *A. brassicicola*, suggesting an essential role for AtMED21 in AtHUB1-mediated immune response against necrotrophic fungi [13]. More recently, it was also shown that AtHUB1 and AtHUB2 are involved in plant defense response to *Verticillium dahliae* toxins through modulating the dynamics of microtubule [46].

In the present study, we examined the involvement of the tomato SlHUB1 and SlHUB2, orthologues of the Arabidopsis AtHUB1 and AtHUB2, in disease resistance against *B. cinerea* and explored the possible molecular mechanisms. We found that virus-induced gene silencing (VIGS) of either *SlHUB1* or *SlHUB2* in tomato plants resulted in increased susceptibility to *B. cinerea* and led to thinner cell wall, increased accumulation of reactive oxygen species (ROS) and callose around the infection sites, demonstrating that both of the SlHUB1 and SlHUB2 are positive regulators of defense response against *B. cinerea* most likely through modulation of cell wall strengthen and ROS balance. Although SlMED21, a subunit of the Mediator complex, interacted with SlHUB1, silencing of *SlMED21* did not affect the disease resistance response to *B. cinerea*, indicating a different mechanism for the function of SlHUB1 and SlHUB2 in defense response against *B. cinerea* from that for AtHUB1 in Arabidopsis.

## Methods

### Plant growth, treatments and disease assays

Tomato (*Solanum lycopersicum*) cv. Suhong 2003 was used for most of the experiments in this study except that cv. MicroTom was used for the whole plant inoculation assays. Seeds were scarified on moist filter paper in Petri dishes for 3 days and then transferred into a mixture of perlite: vermiculite: plant ash (1:6:2). Tomato plants were grown in a growth room under fluorescent light (200 μE m^2^ s^−1^) at 22 ~ 24 °C with 60 % relative humidity in a 14 h light/10 h dark regime. For analysis of gene expression, 4-week-old tomato plants were treated by foliar spraying with 10 μM methyl jasmonate (MeJA), 100 μM 1-amino cyclopropane-1-carboxylic acid (ACC), 100 μM SA or water as a control and samples were collected at indicated time points after treatment.

Inoculation of *B. cinerea* was performed using two different methods, whole plant inoculation and detached leaf inoculation, as previously described [47–49]. Briefly, *B. cinerea* was grown on 2 × V8 agar (36 % V8 juice, 0.2 % CaCO_3_ and 2 % agar) at 22 °C and spores were collected and resuspended in 1 % maltose buffer to 2 × 10^5^ spores/mL for the whole plant inoculation and 1 × 10^5^ spores/mL for the detached leaf inoculation. The concentrations of spore suspension were widely used in previously reported studies [47–50]. In the whole plant inoculation assays, 4-week-old plants were inoculated by foliar spraying with spore suspension or buffer. In the detached leaf inoculation assays, fully expanded leaves were inoculated by dropping 5 μL of spore suspension onto leaf surface. The inoculated leaves and plants were kept in a humidity condition by covering with plastic film in trays or tans at 22 °C to facilitate disease development. Leaf samples were collected from the whole plant inoculation assays at different time points after inoculation for analysis of gene expression and *in planta* fungal growth. Fungal growth was measured by qRT-PCR analyzing the transcript of *B. cinerea ActinA* gene as a growth indicative [51] using a pair of primers BcActin-F and BcActin-R (Additional file 1). Disease in the detached leaf inoculation assays was estimated by measuring the lesion sizes.

Disease assays for *Pst* DC3000 were done as described previously [13, 48, 52]. *Pst* DC3000 was grown overnight in King’s B liquid medium and resuspended in 10 mM MgCl_2_ at OD_600_ = 0.0002. Four-week-old plants were vacuum infiltrated with bacteria suspensions and then kept in a growth chamber with high humidity. For measurement of bacterial growth curve, leaf punches from six individual plants were surface sterilized in 70 % ethanol for 10 s, homogenized in 200 μL of 10 mM MgCl_2_, diluted in 10 mM MgCl_2_, and plated on KB agar plates containing 100 μg/mL rifampicin. Colonies were counted after incubation at 28 °C for 3 days.

### Cloning of *SlHUB1*, *SlHUB2* and *SlMED21*

Rapid amplification of cDNA end (RACE) experiments were carried out using the SMARTer RACE cDNA Amplification Kit (Clontech, Mountain View, CA, USA) with nested primers (Additional file 1) to obtain the 5’ end sequence information. The RACE products were cloned by T/A cloning into pMD19-T vector (Takara, Dalian, China) and sequenced. Based on the sequencing results, pairs of gene-specific primers were designed (Additional file 1) and the full-length cDNAs of *SlHUB1*, *SlHUB2* and *SlMED21* were amplified and cloned into vector pMD19-T, yielding plasmids pMD19-SlHUB1, pMD19-SlHUB2 and pMD19-SlMED21, respectively. These plasmids were confirmed by sequencing and used for all experiments described below.

#### Construction of vectors and VIGS assays

Fragments of 300-400 bp in sizes for *SlHUB1*, *SlHUB2* and *SlMED21* were amplified using gene-specific primers (Additional file 1) from pMD19-SlHUB1, pMD19-SlHUB2 and pMD19-SlMED21, respectively, and were cloned into pTRV2 vector [53], yielding pTRV2-*SlHUB1*, pTRV2-*SlHUB2* and pTRV2-*SlMED21*. These constructs were then introduced into *Agrobacterium tumefaciens* strain GV3101 by electroporation using GENE PULSER II Electroporation System (Bio-Rad Laboratories, Hercules, CA, USA). Agrobacteria carrying pTRV2-*GUS* (as a negative control), pTRV2-*SlHUB1*, pTRV2-*SlHUB2* or pTRV2-*SlMED21* were grown in YEP medium (50 μg/mL rifampicin, 50 μg/mL kanamycin and 25 μg/mL gentamicin) for 24 h with continuous shaking at 28 °C. Cells were centrifuged and resuspended in infiltration buffer (10 mM MgCl_2_, 10 mM MES, 200 μM acetosyringone, pH5.7). Agrobacteria carrying pTRV2-*GUS*, pTRV2-*SlHUB1*, pTRV2-*SlHUB2* or pTRV2-*SlMED21* were mixed with agrobacteria carrying pTRV1 in a ratio of 1:1 and adjusted to OD_600_ = 1.5. The mixed agrobacteria suspension was infiltrated into the abaxial surface of 2-week-old seedlings using a 1 mL needleless syringe. Efficiency of the silencing protocol was examined using phytoene desaturase (PDS) gene as a marker of silencing in tomato plants according to the protocol described previously [53].

### Purification of SlHUB1 and SlHUB2 protein

The coding sequences of *SlHUB1* and *SlHUB2* were amplified using gene-specific primers (Additional file 1) and cloned into pET-32a (NovaGen, Madison, WI, USA) at *Not*I and *Xho*I sites. Meanwhile, truncated mutants SlHUB1^ΔRING^ and SlHUB2^ΔRING^ with deletion of the RING domain in SlHUB1 and SlHUB2, respectively, were amplified using gene-specific primers (Additional file 1) and cloned into pET-32a at *Not*I and *Xho*I sites. The SlHUB1, SlHUB2, SlHUB1^ΔRING^ and SlHUB2^ΔRING^ fusion proteins were expressed in the *E. coli* Rosetta cells (Novagen, Madison, WI, USA) and induced by 1 mM isopropyl-a-thiogalactoside at 30 °C for 4-6 h. The His-tagged SlHUB1, SlHUB2, SlHUB1^ΔRING^ and SlHUB2^ΔRING^ fusion proteins were purified using Ni-NTA His-Bind Resin following the manufacturer’s protocols (Merck BioSciences, Nottingham, UK). The purified proteins were refolded by dialysis in a refolding buffer (50 mM Tris–HCl, 1 mM DTT, 0.5 M NaCl, 0.5 % Triton-X-100, 1 mM PMSF, 4 M urea, pH8.0) at 4 °C for 2 days. Protein concentration was determined with the Bio-Rad protein assay kit (Bio-Rad, Hercules, CA, USA).

### *In vitro* histone monoubiquitination assay

Assays for *in vitro* monoubiquitination were performed as described previously [37]. Briefly, the refolded proteins were incubated with 0.1 μg E1 (BostonBiochem, Cambridge, MA, USA), 0.2 μg Rad6 (BostonBiochem, Cambridge, MA, USA), 10 μg ubiquitin proteins (Merck BioSciences, Nottingham, UK) and 1 μg recombinant H2B (New England Biolabs, Ipswitch, MA, USA) in 30 μL buffer (5 mM MgCl_2_, 4 mM ATP, 50 mM Tris–HCl, 2 mM DTT). Reactions were incubated at 37 °C for 3 h and then terminated by adding SDS-PAGE loading buffer, followed by separation on a 12.5 % SDS-PAGE. Signals were detected by immunoblotting using anti-ubiquitin antibody (Merck BioSciences, Nottingham, UK), followed by chemiluminescence with the ECL kit (Thermo Fisher Scientific, Waltham, MA, USA) according to the manufacture’s recommendations.

### Yeast two-hybrid assays

Interactions between SlHUB1 or SlHUB2 and SlMED21 were examined using the Matchmaker Gold Yeast Two-Hybrid System according to the manufacturer’s instructions (Clontech, Mountain View, CA, USA). The coding sequences of *SlHUB1*, *SlHUB2* and *SlMED21* were amplified using gene-specific primers (Additional file 1) from pMD19-SlHUB1, pMD19-SlHUB2 and pMD19-SlMED21, respectively, and cloned into pGADT7 and pGBKT7 vectors. The resultant plasmids were transformed into yeast strains Y187 and Y2HGold and confirmed by colony PCR. The transformed yeasts were cultivated on SD/Trp^−^ and SD/Trp^−^His^−^ medium for 3 days at 30 °C, followed by addition of X-α-Gal (5-Bromo-4chloro-3-indolyl-a-D-galactopyranoside). Interactions between SlHUB1/SlHUB2 and SlMED21 were evaluated according to the growth situation of the transformed yeast cells on the SD/Trp^−^His^−^ medium and production of blue pigments after the addition of X-α-Gal. Co-transformation of pGBKT7-53 and pGADT7-T were as a positive control.

### Detection of ROS accumulation

Detection of H_2_O_2_ and superoxide anion in leaf tissues were conducted according to previously described procedures [50]. For staining of H_2_O_2_, samples were dipped into 3, 3-diaminobenzidine (DAB) (Sigma-Aldrich, St. Louis, MO, USA) solution (1 mg/mL, pH 3.8) and incubated for 8 h in the dark at room temperature. For staining of superoxide anion, leaves were dipped into the 10 mM potassium phosphate buffer (pH 7.5) containing 10 mM NaN_3_ and 0.1 % nitroblue tetrazolium (NBT) (Sigma-Aldrich, St. Louis, MO, USA) for 1 h at room temperature. To remove the chlorophyll, leaves were placed into 95 % ethanol and boiled in a water bath, followed by several changes of the solution. The leaves were then maintained in 50 % ethanol and the accumulation of H_2_O_2_ and superoxide anion in leaves was photographed using a digital camera.

### Callose staining

Callose staining was performed as describe previously [54]. Leaves were cleared in alcoholic lactophenol solution for 30 min at 65 °C, transferred to fresh alcoholic lactophenol solution and then incubated overnight at room temperature. Cleared leaves were rinsed briefly in 50 % ethanol, then water, and stained with 0.01 % aniline blue (Sigma-Aldrich, St. Louis, MO, USA) in 150 mM sodium phosphate buffer for 45 min in the dark, followed by washing with fresh sodium phosphate buffer. The leaf samples were examined under a Leica CTR5000 microscopy (Leica Microsystems, Hong Kong, China) with an excitation filter of 365 ± 25 nm, a 400-nm dichroic mirror and a 450-nm longpass emission filter and callose deposits were visualized as light blue spots against a dark blue background [54]. Pictures showing callose deposits surrounding the infection sites were taken at a similar exposure. The quantification of callose in inoculated tissue was done using ImageJ software (http://rsb.info.nih.gov/ij/download.html). The same threshold defining a fluorescent and a nonfluorescent area was used for all the infected samples and controls, respectively. The area (in percentage) showing fluorescence in the infected tissue above the mock-inoculated control was calculated.

### Transmission electron microscopy

Leaves from 4-week-old plants were collected and fixation was performed using the microwave method as described previously [13]. Briefly, the samples were immersed in primary fixation buffer (2 % paraformaldehyde and 2.5 % glutaraldehyde in 0.1 M potassium phosphate buffer, pH 6.8) overnight, followed by a secondary fixation with reduced osmium (1 % OsO_4_ and 1.5 % K_3_Fe(CN)_6_) after washing with 0.1 M potassium phosphate buffer. The fixed leaf samples were dehydrated by an ethanol series and propylene oxide and then embedded in Epon812 resin. Ultra-thin sections were stained by uranyl acetate and alkaline lead citrate for 15 min, respectively, and observed under a Hitachi H-7650 transmission electron microscope (Hitachi, Tokyo, Japan).

### Real-time quantitative RT (qRT)-PCR analysis of gene expression

Total RNA was extracted using TRIzol reagent (Invitrogen, Shanghai, China) and treated with RNase-free DNase (TaKaRa, Dalian, China) to erase any genomic DNA in the RNA samples according to the manufactures’ instructions. For qRT-PCR analysis, RNA samples were reverse transcribed with oligo(dT) using PrimeScript reagent kit with gDNA eraser (TaKaRa, Dalian, China). qRT-PCR was performed on a CFX96 Real-Time PCR detection system (BioRad, Hercules, CA, USA) using SYBR Premix Ex TaqTM kits (TaKaRa, Dalian, China). A tomato *Actin1* gene (*SlActin*) was used as the internal standard for normalizing the qRT-PCR data. Three independent biological replicates were done. The relative expression levels were calculated using the 2^-ΔΔCT^ method. Primers used for qRT-PCR are listed in Additional file 1.

### Statistical analysis

All experiments were performed in triplicates and data are shown as mean ± SD from three independent experiments. Data were subjected to statistical analysis according to the Student’s *t*-test and the probability values of *p* < 0.05 were considered as significant difference.

## Results

### Identification of tomato SlHUB1 and SlHUB2

**Fig. 1:**
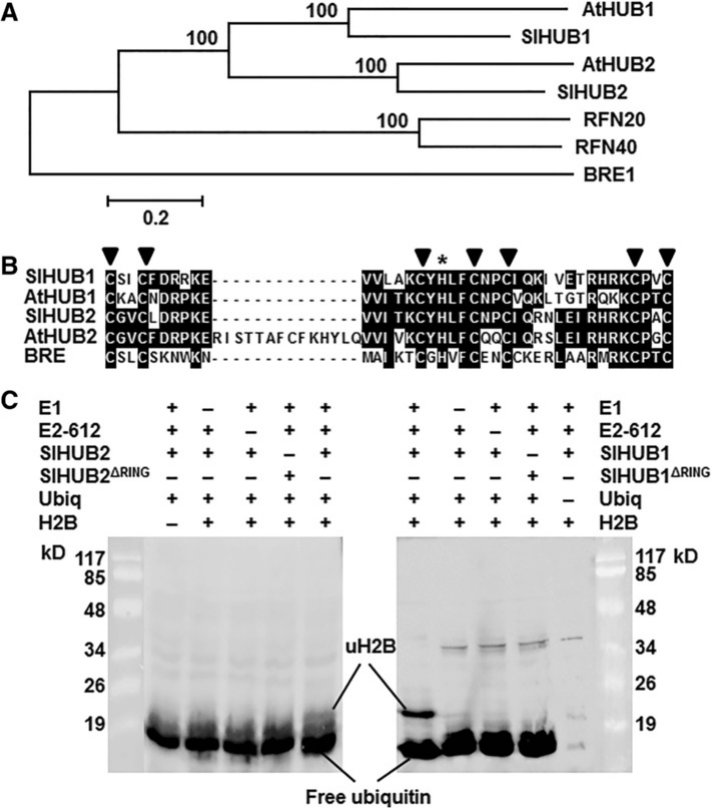
SlHUB1 and SlHUB2 are functional histone H2B monoubiquitination E3 ligases. **a** Phylogenetic tree analysis of SlHUB1 and SlHUB2 with yeast BRE (GenBank accession No. Q07457), Arabidopsis AtHUB1 (Q8RXD6) and AtHUB2 (NP_564680), and human RFN20 (NP_062538) and RFN40 (NP_001273501). Sequence alignment was performed using ClustalX 1.81 program and phylogenic tree was created and visualized using MEGA 6.06. **b** Amino acid alignments of the SlHUB1 and SlHUB2 RING domains with RING domains of the Arabidopsis AtHUB1 and AtHUB2 and yeast BRE. Filled triangles indicate the conserved cysteine residues, while asterisk indicates conserved histidine residue. **c** Recombinant SlHUB1 (right) and SlHUB2 (left) proteins have histone H2B monoubiquitination activity *in vitro*. Recombinant SlHUB1 and SlHUB2 and their mutants SlHUB1^ΔRING^ and SlHUB2^ΔRING^ were incubated with E1 enzyme, E2 enzyme (Rad6), H2B substrate and ubiquitin, separated on SDS-PAGE and detected by Western blotting using anti-ubiquitin antibody. The absences of each one of H2B, E1, E2 or ubiquitin were included as negative controls

To characterize putative orthologues of Arabidopsis AtHUB1 and AtHUB2 in tomato, we performed BlastP searches against the tomato genomic database (ITAG release 2.31) using the amino acid sequences of AtHUB1 and AtHUB2 proteins as queries and obtained three predicted loci (Solyc11g013370, Solyc01g006030 and Solyc01g006040) with high levels of sequence similarity or identity. Further analyses led to the identification of the locus Solyc11g013370 as putative *SlHUB1* whereas both of the predicted loci Solyc01g006030 and Solyc01g006040 as putative *SlHUB2*. The full-length cDNAs of *SlHUB1* and *SlHUB2* were cloned and confirmed by sequencing. *SlHUB1* encodes an 847 amino acid protein while *SlHUB2* encodes an 883 amino acid protein. Sequence alignment and phylogenetic tree analysis revealed that the tomato SlHUB1 and SlHUB2 show 56–76 % of identity to yeast BRE1 [55], human RNF20 and RNF40 [56] and Arabidopsis AtHUB1 and AtHUB2 [37] (Fig. 1a), and both of them contain a conserved C3HC4 RING domain at C-terminus (position at 795–833 aa for SlHUB1 and 831–869 aa for SlHUB2) (Fig. 1b). Therefore, the cloned *SlHUB1* and *SlHUB2* are putative Arabidopsis AtHUB1 and AtHUB2 orthologues in tomato.

### SlHUB1 and SlHUB2 had histone H2B monoubiquitination activity *in vitro*

To determine whether SlHUB1 and SlHUB2 have histone H2B monoubiquitination E3 ligase activity, the *SlHUB1* and *SlHUB2* were expressed prokaryotically and the recombinant His-tagged SlHUB1 and SlHUB2 proteins were purified. To examine the importance of the RING domain in E3 ligase activity, truncated mutants of SlHUB1 and SlHUB2, SlHUB1^ΔRING^ and SlHUB2^ΔRING^, in which the RING domains were deleted, were also generated (Fig. 1c). In the presence of histone 2B, E1 enzyme, E2 (Rad6) enzyme and ubiquitin [37, 57], both of the recombinant SlHUB1 and SlHUB2 could ubiquitinate the histone 2B, as revealed by the two bands of ~8 Kd and ~23 kD, responsible for free ubiquitin and ubiquitinated histone, respectively, that were reactive to ubiquitin-specific antibody, while only one ~8 Kd bind, referring to free ubiquitin in the reactions, was detected in the absence of E1, E2, or SlHUB1 or SlHUB2 (Fig. 1c). The truncated mutants, SlHUB1^ΔRING^ and SlHUB2^ΔRING^, did not show E3 ligase activity in the reactions (Fig. 1c). These results indicate that both of SlHUB1 and SlHUB2 act as functional histone H2B monoubiquitination E3 ligases and that the RING domains in SlHUB1 and SlHUB2 are essential to their histone H2B monoubiquitination activity.

### Expression of *SlHUB1* and *SlHUB2* was induced by pathogens and hormones

**Fig. 2:**
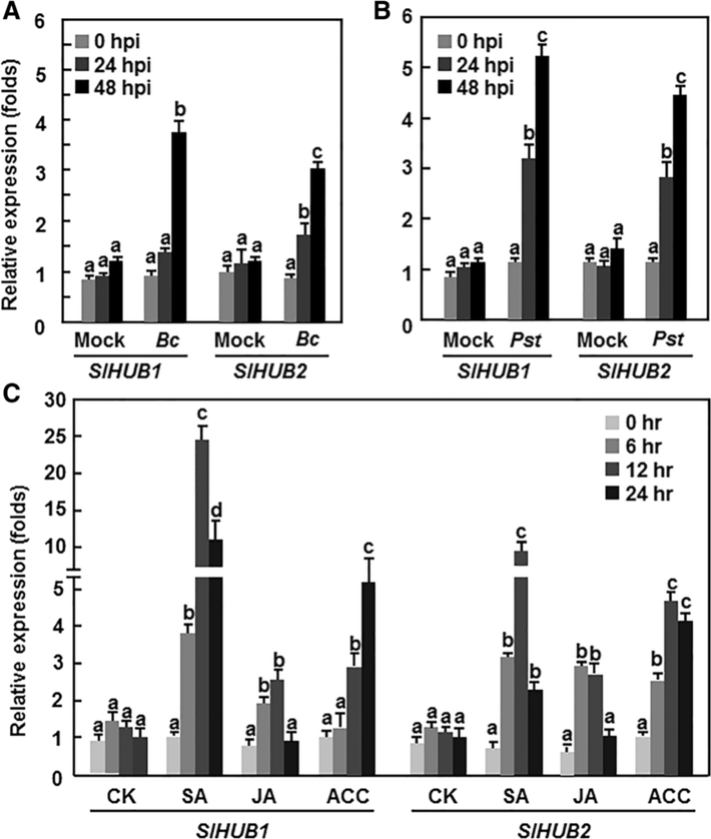
Expression of *SlHUB1* and *SlHUB2* in responses to pathogens and defense signaling-related hormones. Four-week-old plants were inoculated by spore suspension of *B. cinerea* (**a**), vacuum-infiltrated by suspension of *Pst* DC3000 (**b**) or treated by foliar spraying with 1 mM SA, 100 μM MeJA, 100 μM ACC solutions or sterilized distill water as a control (**c**). Leaf samples were collected at indicated time points after treatment. Relative expression was shown as folds of the actin transcript values. Data presented are the means ± SD from three independent experiments and different letters above the columns indicate significant differences at *p* < 0.05 level

To explore the possible roles of *SlHUB1* and *SlHUB2* in tomato disease resistance, we first analyzed the expression patterns of *SlHUB1* and *SlHUB2* in response to pathogens and defense signaling-related hormones. The expression of *SlHUB1* and *SlHUB2* in mock-inoculated plants maintained unchanged during the experimental period (Fig. 2a). However, the expression levels of *SlHUB1* and *SlHUB2* increased after *B. cinerea* infection, showing approximately 4-fold increases over that in mock-inoculated plants at 48 h after inoculation (Fig. 2a). Similar expression dynamics of *SlHUB1* and *SlHUB2* were also observed in plants inoculated with *Pst* DC3000 but the induction was much faster than that in *B. cinerea*-inoculated plants (Fig. 2b). The expression levels of *SlHUB1* and *SlHUB2* increased markedly at 24 h and showed further increases at 48 h after inoculation, leading to ~3-fold of increases over that in the mock-inoculated plants, although increases of *SlHUB1* and *SlHUB2* expression were also observed at 48 h in mock-inoculated plants (Fig. 2b). The expression of *SlHUB1* and *SlHUB2* was induced at different levels by the defense signaling-related hormones. SA drastically induced the expressions of *SlHUB1* and *SlHUB2*, showing ~10-fold and 6.5-fold of increases for *SlHUB1* and *SlHUB2*, respectively, over those in the control plants at 12 h after treatment (Fig. 2c). The expression level of *SlHUB1* in ACC-treated plants showed 4.2-fold of increase at 24 h after treatment over that in the control plants whereas the expression levels of *SlHUB2* were increased significantly in the ACC-treated plants, giving 2.1-fold and 3.2-folds of increases over those in the control plants at 12 and 24 h after treatment (Fig. 2c). However, no significant induction in the expression levels of *SlHUB1* and *SlHUB2* was observed after treatment with JA, as compared with the expression in the control plants (Fig. 2c). These results indicate that the expression of *SlHUB1* and *SlHUB2* can be induced by *B. cinerea* and *Pst* DC3000 and by defense signaling-related hormones, such as SA, JA and ACC.

### Silencing of *SlHUB1* or *SlHUB2* resulted in increased susceptibility to *B. cinerea*

**Fig. 3:**
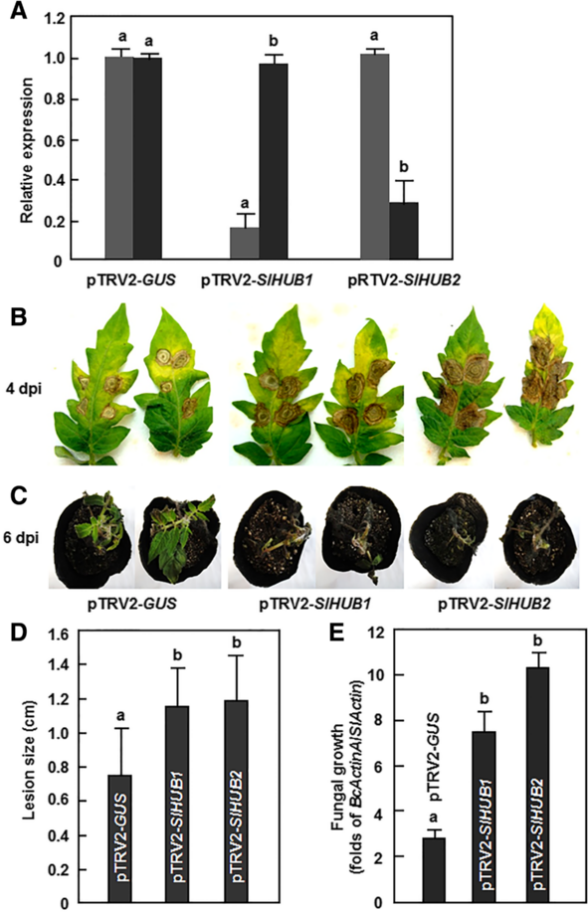
Silencing of *SlHUB1* and *SlHUB2* resulted in reduced resistance to *B. cinerea*. Two-week-old seedlings were infiltrated with agrobacteria carrying pTRV2-*SlHUB1*, pTRV2-*SlHUB2* or pTRV2-*GUS* constructs and disease assays were carried out at 4 weeks after VIGS infiltration. **a** Silencing efficiency of *SlHUB1* and *SlHUB2* in VIGS construct-infiltrated plants. The transcript levels of *SlHUB1* or *SlHUB2* in pTRV2-*SlHUB1* or pTRV2-*SlHUB2*-infiltrated plants were analyzed by qRT-PCR and compared to that in pTRV2-*GUS*-infiltrated plants, which was set 1. **b**, **d** Disease phenotype (**b**) and lesion size (**d**) on detached leaves of pTRV2-*SlHUB1*, pTRV2-*SlHUB2* or pTRV2-*GUS*-infiltrated plants after drop-inoculation with *B. cinerea*, respectively. Photographs were taken at 4 days post-inoculation (dpi). Lesion sizes were measured at 4 dpi and on a minimum of 30 leaves in each experiment. **c**, **e** Disease phenotype (**c**) and fungal growth (**e**) of pTRV2-*SlHUB1*, pTRV2-*SlHUB2* or pTRV2-*GUS*-infiltrated plants after spraying with *B. cinerea*, respectively. Photographs were taken at 6 dpi. Growth of *B. cinerea in planta* was measured at 3 dpi by analyzing the transcript level of *BcActinA* gene with the *SlActin* gene as an internal control. Data presented are the means ± SD from three independent experiments and different letters above the columns indicate significant differences at *p* < 0.05 level

To explore the possible function of *SlHUB1* and *SlHUB2* in disease resistance to *B. cinerea*, we used the TRV-based gene silencing system to knockdown the expression levels of *SlHUB1* or *SlHUB2* in tomato plants and compared the phenotype between the silenced and the control plants after infection with *B. cinerea*. In our standard VIGS experiments, the VIGS procedure efficiency was confirmed as >80 %, as judged by the appearance of bleaching phenotype in the pTRV-*PDS*-infiltrated plants (Additional file 2). Using the standard VIGS procedure, the silencing efficiency was estimated, by comparison of the transcript levels in the pTRV2-*SlHUB1*- or pTRV2-*SlHUB2*-infiltrated plants with those in the pTRV2-*GUS*-infiltrated plants, respectively, to be ~80 % for *SlHUB1* and ~70 % for *SlHUB2* (Fig. 3a). Importantly, the transcript levels of *SlHUB2* and *SlHUB1* in the pTRV2-*SlHUB1*- and pTRV2-*SlHUB2*-infiltrated plants, respectively, were comparable to those in the pTRV2-*GUS*-infiltrated plants, indicating that silencing of *SlHUB1* or *SlHUB2* did not affect the expression of another one. Only when VIGS procedure efficiency was >80 % in the same batch and the silencing efficiency for the target genes (SlHUB1 or SlHUB2) was >70 %, the pTRV-*SlHUB1*- or pTRV-*SlHUB2*-infiltrated plants were used for all experiments at 4 weeks after VIGS infiltration.

We first examined whether silencing of SlHUB1 or SlHUB2 affected the disease resistance against *B. cinerea* in tomato. Detached leaf assays were first performed with fully expanded leaves collected from the pTRV2-*SlHUB1*- and pTRV2-*SlHUB2*-infiltrated plants. Under our disease assay conditions, typical disease symptoms, e.g. necrotic lesions, were observed in the leaves from the pTRV2-*SlHUB1*-, pTRV2-*SlHUB2*- and pTRV2-*GUS*-infiltrated plants at 2 dpi; however, the lesions in the leaves from the pTRV2-*SlHUB1*- or pTRV2-*SlHUB2*-infiltrated plants expanded rapidly and were larger than those in the pTRV2-*GUS*-infiltrated plants (Fig. 3b). At 4 dpi, the lesion sizes in the leaves from the pTRV2-*SlHUB1*- and pTRV2-*SlHUB2*-infiltrated plants were measured as 11.5 mm and 12.1 mm in average, respectively, leading to 66.7 % and 75.4 % of increases over that in the pTRV-*GUS*-infiltrated plants (average of 6.9 mm) (Fig. 3d). Whole plant inoculation assays were also performed to confirm the results obtained from the detached leaf inoculation assays. As shown in Fig. 3c, the pTRV2-*SlHUB1*- or pTRV2-*SlHUB2*-infiltrated cv. MicroTom plants suffered much serious disease as compared with the pTRV2-*GUS*-infiltrated cv. MicroTom plants and, at 6 days after inoculation, approximately 90 % of the pTRV2-*SlHUB1*- or pTRV2-*SlHUB2*-infiltrated plants died while most of the pTRV2-*GUS*-infiltrated plants were still alive. Quantification of *in planta* fungal growth by qRT-PCR analysis of the transcript of the *B. cinerea BcActinA* gene as indicative of the growth rate showed that the fungal biomass, as judged by the folds of *BcActinA*/*SlActin* in the pTRV2-*SlHUB1*- and pTRV2-*SlHUB2*-infiltrated plants was significantly higher than that in the pTRV2-*GUS*-infiltrated plants, leading to 2.3 and 3.4 folds of increases, respectively (Fig. 3e). Collectively, these data indicate that silencing of either *SlHUB1* or *SlHUB2* attenuated the disease resistance in tomato against *B. cinerea* and thus demonstrate that both of *SlHUB1* and *SlHUB2* are required for resistance against *B. cinerea* in tomato.

### SlMED21 interacted with SlHUB1 but silencing of *SlMED21* did not affect the resistance to *B. cinerea*

**Fig. 4:**
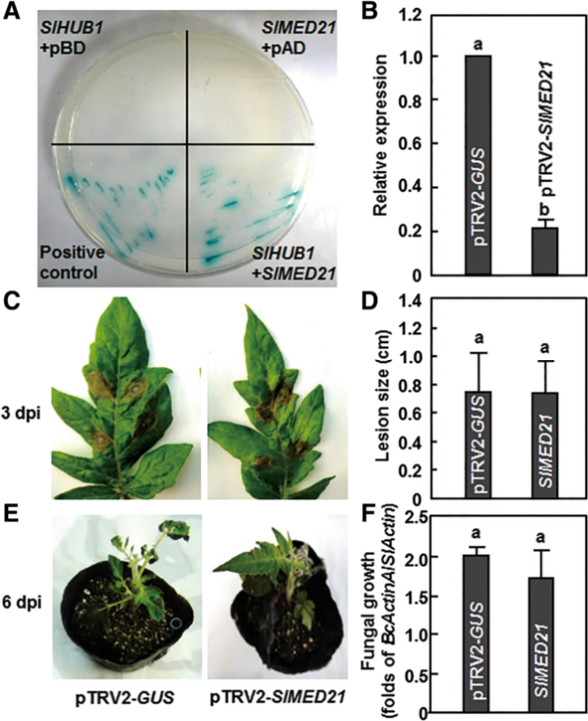
SlMED21 interacts with SlHUB1 but did not affect the resistance to *B. cinerea*. **a** SlMED21 interacted with SlHUB1 in yeast two-hybrid assay. Yeasts carrying the *SlMED21* in the prey vector and the *SlHUB1* in the bait prey vector were assayed for growth on selective medium (SD/Leu^−^ Trp^−^ Ade^−^ His^−^) and β-galactosidase activity after addition of X-α-Gal. The positive control pGADT7-T + pGBKT7-53 and other indicated combinations between empty vector and SlHUB1/SlMED21 were assayed in parallel. **b** Silencing efficiency of *SlMED21* in VIGS construct-infiltrated plants. The silencing efficiency was calculated by comparing the transcript levels of *SlMED21* in pTRV2-*SlMED21*-infiltrated plants to that in pTRV2-*GUS*-infiltrated plants, which were set as 1. **c** Disease symptom on detached leaves at 3 dpi. **d** Disease phenotype on whole plants at 6 dpi, respectively. **e** Lesion sizes on selected leaves in detached leaf inoculation assays at 3 dpi. Lesion sizes were measured on a minimum of 30 leaves in each experiment. **f** Growth of *B. cinerea* in inoculated plants from the whole plant inoculation experiments at 3 dpi. Relative fungal growth was shown as folds of transcript levels of *BcActin* compared to *SlActin*. Data presented are the means ± SD from three independent experiments and different letters above the columns indicate significant differences at *p* < 0.05 level

In Arabidopsis, AtMED21 was shown to interact strongly with AtHUB1 and have a function in the defense response against necrotrophic fungal pathogens [13]. We therefore cloned *SlMED21*, a tomato orthologue of Arabidopsis *AtMED21* gene (Additional file 3), and examined whether tomato SlMED21 also can interact with SlHUB1 or SlHUB2 and thus play a role in resistance to *B. cinerea*. In our yeast two-hybrid assays, a strong interaction between SlMED21 and SlHUB1 was detected when the SlHUB1 in the bait vector (pBD-SlHUB1) and SlMED21 in the prey vector (pAD-SlMED21) were co-expressed in yeast. When co-expressed with the pAD or pBD empty vector, neither SlHUB1 nor SlMED21 activated the transcription of reporter genes, indicating that SlHUB1 or SlMED21 does not have autoactivation activity. In addition, no significant interaction between SlHUB2 and SlMED21 was detected (data not shown). We next examined whether SlMED21 plays a role in resistance against *B. cinerea* by the VIGS-based functional analysis. The silencing efficiency of *SlMED21* under our experimental condition was estimated to be ~80 % (Fig. 4b), as examined by qRT-PCR analysis of the transcript level of *SlMED21* in the pTRV2-*SlMED21*-infiltrated plants compared with that in the pTRV2-*GUS*-infiltrated plants. The disease severity on leaves from pTRV2-*SlMED21*-infiltrated plants were comparable to that from pTRV2-*GUS*-infiltrated plants (Fig. 4c and e). The lesion sizes and the rate of fungal growth in leaves of the pTRV2-*SlMED21*-infiiltrated plants were also similar to those in leaves of the pTRV2-*GUS*-infiltrated plants (Fig. 4d and f). These results indicate that silencing of *SlMED21* did not affect the resistance of tomato plants against *B. cinerea*, although SlMED21 did interact with SlHUB1.

### Silencing of SlHUB1 but not SlHUB2 and SlMED21 affected resistance to Pst DC3000

**Fig. 5:**
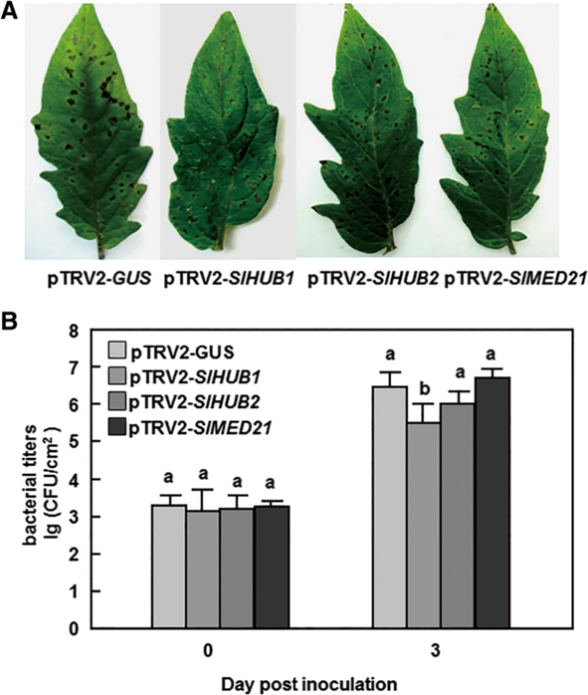
Silencing of *SlHUB1* resulted in increased resistance to *P. syringae* pv. *tomato* DC3000. Two-week-old seedlings were infiltrated with agrobacteria carrying pTRV2-*SlHUB1*, pTRV2-*SlHUB2*, pTRV2-*SlMED21* or pTRV2-*GUS* conducts and disease assays were performed by vacuum infiltrating with *Pst* DC3000 at 4 weeks after VIGS infiltration. **a** Representative symptom of disease caused by *Pst* DC3000 at 4 dpi. **b** Bacterial growth in inoculated leaves of pTRV2-*SlHUB1*-, pTRV2-*SlHUB2*-, pTRV2-*SlMED21*- or pTRV2-*GUS*-infiltrated plants. Leaf samples were collected at 0 and 4 days after inoculation and bacterial growth was measured. Data presented are the means ± SD from three independent experiments and different letters above the columns indicate significant differences at *p* < 0.05 level

To explore the possible involvement of *SlHUB1*, *SlHUB2* and *SlMED21* in defense response against other pathogens, we further examined whether silencing of SlHUB1, SlHUB2 or SlMED21 affects the resistance to *Pst* DC3000. In our experiments, necrotic lesions were observed in the inoculated leaves of the pTRV2-*SlHUB1*-, pTRV2-*SlHUB2*-, pTRV2-*SlMED21*- and pTRV2-*GUS*-infiltrated plants. However, the lesions on leaves of the pTRV2-*SlHUB1*-infiltrated plants were less and smaller than those in the pTRV2-*GUS*-infiltrated plants, while no significant difference was observed between the pTRV2-*SlHUB2*-, pTRV2-*SlMED21*- and pTRV2-*GUS*-infiltrated plants after vacuum inoculated with *Pst* DC3000 (Fig. 5a). At 3 dpi, the bacterial population in the inoculated leaves of the pTRV2-*SlHUB1*-infiltrated plants (5.49 × 10^5^ cfu/cm^2^) showed about 10-fold lower to that in the pTRV2-*GUS*-infiltrated plants (6.45 × 10^6^ cfu/cm^2^) while there are no significant difference in bacterial growth in the inoculated leaves of the pTRV2-*SlHUB2*- (6.01 × 10^6^ cfu/cm^2^), pTRV2-*SlMED21*- (6.69 × 10^6^ cfu/cm^2^) and pTRV2-*GUS*-infiltrated plants (Fig. 4b). These results indicate that silencing of *SlHUB1* resulted in increased resistance against *Pst* DC3000, but silencing of either *SlHUB2* or *SlMED21* did not affect the resistance against *Pst* DC3000, implying an involvement of SlHUB1 in defense response to *Pst* DC3000.

### Silencing of *SlHUB1* and *SlHUB2* resulted in reduced cell wall thickness through modulating the phenylpropanoid pathway

**Fig. 6:**
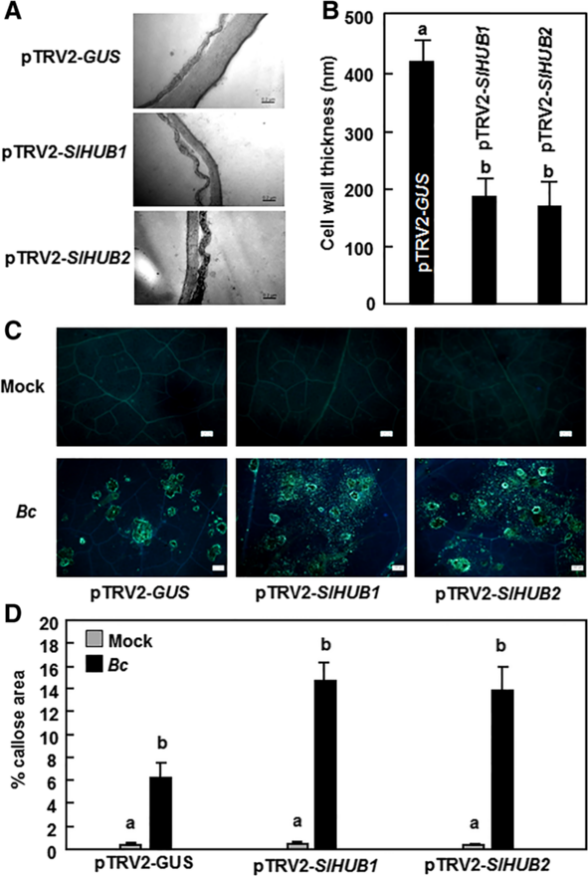
Silencing of *SlHUB1* and *SlHUB2* resulted in reduced cell wall thickness but increased callose accumulation after *B. cinerea* infection*.* Two-week-old seedlings were infiltrated with agrobacteria carrying pTRV2-*SlHUB1*, pTRV2-*SlHUB2*, pTRV2-*SlMED21* or pTRV2-*GUS* constructs. **a**, **b** Representative TEM photos showing the cell wall (**a**) and the thickness of cell wall (**b**) in pTRV2-SlHUB1-, pTRV2-SlHUB2- or pTRV2-GUS-infiltrated plants. Leaf samples were collected for TEM assays at 4 weeks after VIGS infiltration. Bars = 200 nm. The data represent mean ± SE from 20 samples. **c** Callose accumulation. The VIGS construct-infiltrated plants were inoculated with *B. cinerea* and at least 6 leaves from 6 individual plants were collected at 0 h and 24 h after inoculation for detection of callose accumulation. Upper row represents callose staining in mock-inoculated leaves whereas lower row represents callose staining in *B. cinerea*-inoculated leaves. Bars = 100 μm. The callose data shown in (**d**) were quantified using an image analysis program as described in Methods

Mutations in the Arabidopsis AtHUB1 were previously found to be involved in the regulation of cell wall thickness and callose deposition [13]. We thus investigated whether SlHUB1 and SlHUB2 act through a similar mechanism in tomato as AtHUB1 in Arabidopsis by examining the differences in the cell walls and callose deposition in leaves of SlHUB1- and SlHUB2-silenced plants. Transmission electron microscopy examination showed that the cell wall in leaves of the pTRV2-*SlHUB1*- or pTRV2-*SlHUB2*-infiltrated plants was significantly thinner than that in the pTRV2-*GUS*-infiltrated plants (Fig. 6a). The thickness of the cell walls in leaves of the pTRV2-*SlHUB1*- or pTRV2-*SlHUB2*- infiltrated plants were measured as 185.6 nm and 168.9 nm, showing 55.8 % and 59.9 % of reduction, respectively, compared with that (421 nm) in the pTRV2-*GUS*-infiltrated plants (Fig. 6b). We compared the callose deposition in the pTRV2-*SlHUB1*- and pTRV2-*SlHUB2*-infiltrated plants with that in the pTRV2-*GUS*-infiltrated plants before and after infection by *B. cinerea*. No significant callose deposition was observed in mock-inoculated plants and no difference in callose deposition was found among pTRV2-*SlHUB1*-, pTRV2-*SlHUB2*- and pTRV2-*GUS*-infiltrated plants (Fig. 6c and d). Infection of *B. cinerea* significantly induced callose deposition in cells surrounding the infection sites in the pTRV2-*SlHUB1*-, pTRV2-*SlHUB2*- and pTRV2-*GUS*-infiltrated plants (Fig. 6c and d); however, callose depositions in the pTRV2-*SlHUB1*- and pTRV2-*SlHUB2*-infiltrated plants were much evident than that in the pTRV2-*GUS*-infiltrated plants (Fig. 6c and d). These data indicate that silencing of either *SlHUB1* or *SlHUB2* resulted in thinner cell wall but triggered more callose deposition in response to *B. cinerea*.

**Fig. 7:**
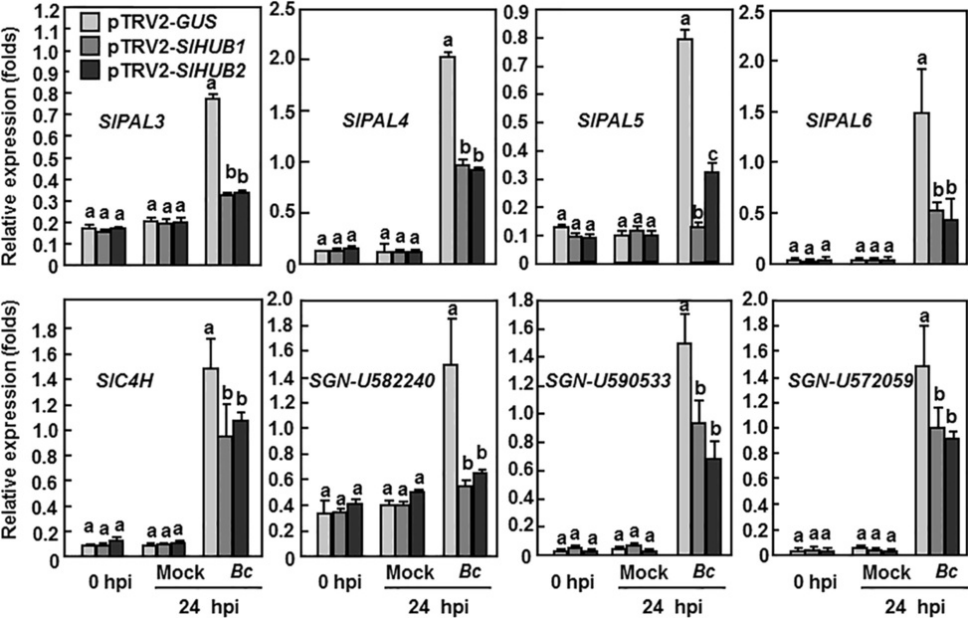
Silencing of *SlHUB1* and *SlHUB2* attenuated the expression of phenylpropanoid pathway-related genes after *B. cinerea* infection*.* Two-week-old seedlings were infiltrated with agrobacteria carrying pTRV2-*SlHUB1*, pTRV2-*SlHUB2*, pTRV2-*SlMED21* or pTRV2-*GUS* constructs and were inoculated with spore suspension of *B. cinerea* at 4 weeks after VIGS infiltration. At least 6 leaves from 6 individual plants were collected at 0 and 24 h after inoculation and used for analysis of gene expression. Data presented are the means ± SD from three independent experiments and different letters above the columns indicate significant differences at *p* < 0.05 level

It is well known that the phenylpropanoid pathway is involved in the cell wall biosynthesis [58]. We therefore analzyed and compared the expression changes of genes coding for phenylalanine ammonia lyase (PAL) [59], cinnamate 4-hydroxylase (C4H) [60] and cinnamoyl alcohol dehydrogenase (CAD) in the pTRV2-*SlHUB1*- and pTRV2-*SlHUB2*-infiltrated plants with that in the pTRV2-*GUS*-infiltrated plants before and after infection by *B. cinerea*. In healthy (0 h after inoculation) and in mock-inoculated plants (24 h after inoculation), the expression levels of tested *SlPALs* (*SlPAL3*, *SlPAL4* and *SlPAL6*), *SlC4H* and three *SlCAD* (*SGN-U582240*, *SGN-U590533*, *SGN-U572059*) genes were comparable among the pTRV2-*SlHUB1*-, pTRV2-*SlHUB2*-, and pTRV2-*GUS*-infiltrated plants (Fig. 7). Notably, the expression level of *SlPAL5* in the pTRV2-*SlHUB1*- and pTRV2-*SlHUB2*-infiltrated plants was significantly reduced by 60–84 % as compared to in the pTRV2-*GUS*-infiltrated plants without infection of *B. cinerea* (Fig. 7). After *B. cinerea* infection, the expression levels of *SlPALs*, *SlC4H* and *SlCADs* were markedly upregulated in the pTRV2-*GUS*-infiltrated plants as compared to those in the mock-inoculated plants; however, the *B. cinerea*-induced expression of *SlPALs*, *SlC4H* and *SlCADs* were significantly decreased by 40–80 % in the pTRV2-*SlHUB1*- and pTRV2-*SlHUB2*-infiltrated plants compared with those in the pTRV2-*GUS*-infiltrated plants (Fig. 7). These data indicate that silencing of either *SlHUB1* or *SlHUB2* attenuated the *B. cinerea*-induced expression of a set of genes in the phenylpropanoid pathway.

### Silencing of *SlHUB1* and *SlHUB2* increased ROS generation upon *B. cinerea* infection

**Fig. 8:**
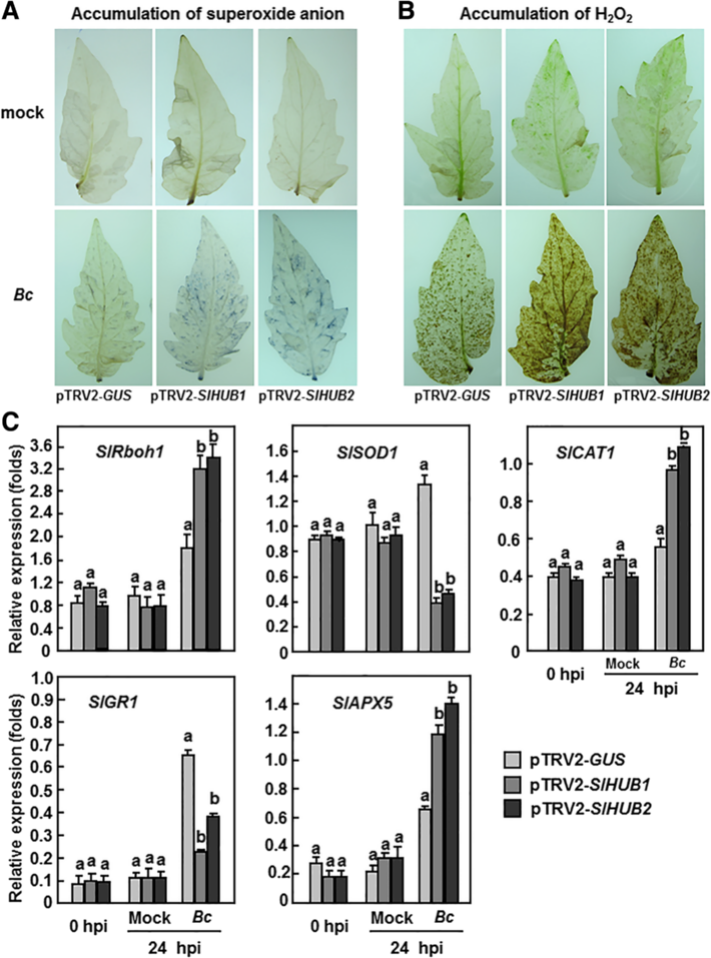
Silencing of *SlHUB1* and *SlHUB2* resulted in accumulation of ROS and affected the expression of ROS generation- and scavenging-related genes after infection with *B. cinerea*. Two-week-old seedlings were infiltrated with agrobacteria carrying pTRV2-*SlHUB1*, pTRV2-*SlHUB2* or pTRV2-*GUS* constructs and were inoculated by spraying with spore suspension of *B. cinerea* or with buffer as mock-inoculation control at 4 weeks after VIGS infiltration. At least 6 leaves from 6 individual plants were collected at 0 (as controls) and 24 h after inoculation. **a** Accumulation superoxide anion. **b** Accumulation of H_2_O_2_. Representative NBT- or DAB-stained leaves are shown and similar results were obtained from repeated experiments. **c** Expression of ROS generation- and scavenging-related genes before and after infection with *B. cinerea*. Relative expression levels were shown as folds of the actin transcript values. Data presented are the means ± SD from three independent experiments and different letters above the columns indicate significant differences at *p* < 0.05 level

Considering that reactive oxygen species (ROS) has been involved in susceptible responses of plants to infection from necrotrophic fungal pathogens such as *B. cinerea* [61], we also examined whether silencing of *SlHUB1* or *SlHUB2* affects the balance of ROS in tomato plants upon infection of *B. cinerea*. In healthy (0 h after inoculation) and in mock-inoculated plants (24 h after inoculation), no significant difference in accumulation of H_2_O_2_ and superoxide anion was detected in leaves of the pTRV-*SlHUB1*-, pTRV-*SlHUB2*- and pTRV-*GUS*-infiltrated plants (Fig. 8a and b), indicating that silencing *SlHUB1* or *SlHUB2* did not affect the accumulation of ROS in tomato. In contrast, at 24 h after inoculation with *B. cinerea*, accumulation of superoxide anion and H_2_O_2_ in leaves of the pTRV2-*SlHUB1*-, pTRV2-*SlHUB2*-, and pTRV2-*GUS*-infiltrated plants was markedly increased, as compared with those in the mock-inoculated plants (Fig. 8a and b). However, the accumulation of ROS in the pTRV2-*SlHUB1*- and pTRV2-*SlHUB2*-infiltrated plants was much evident than that in the pTRV2-*GUS*-infiltrated plants (Fig. 8a and b). We further compared the expression changes of genes involved in ROS generating and scavenging system. The expression levels of a ROS generation-related gene, *SlRboh1* [62, 63], and several ROS scavening-related genes such as *SlSOD1* (superoxide dismutase), *SlCAT1* (catalase), *SlGR1* (glutathione reductase) and *SlAPX5* (ascorbate peroxidase) were comparable among the pTRV2-*SlHUB1*-, pTRV2-*SlHUB2*-, and pTRV2-*GUS*-infiltrated healthy and mock-inoculated plants (Fig. 8a and b). Two distinct expression patterns for these genes were observed in the pTRV2-*SlHUB1*-, pTRV2-*SlHUB2*-, and pTRV2-*GUS*-infiltrated plants after inoculation with *B. cinerea* (Fig. 8a and b). At 24 h after inoculation with *B. cinerea*, the expression levels of *SlRboh1*, *SlCAT1* and *SlAPX5* were significantly increased by 75-120 %, whereas the expression levels of *SlSOD1* and *SlGR1* were markedly reduced by 1 ~ 2-fold in the pTRV2-*SlHUB1*- and pTRV2-*SlHUB2*-infiltrated plants, as compared to the corresponding levels in the pTRV2-*GUS*-infiltrated plants (Fig. 8a and b). These data indicate that silencing of either *SlHUB1* or *SlHUB2* could potentiate the generation and accumulation of ROS through affecting the expression of genes associated with the ROS generating and scavenging system upon *B. cinerea* infection.

### Silencing of *SlHUB1* or *SlHUB2* attenuated the JA/ET-mediated signaling and defense response but activated the SA-mediated signaling and defense response upon *B. cinerea* infection

**Fig. 9:**
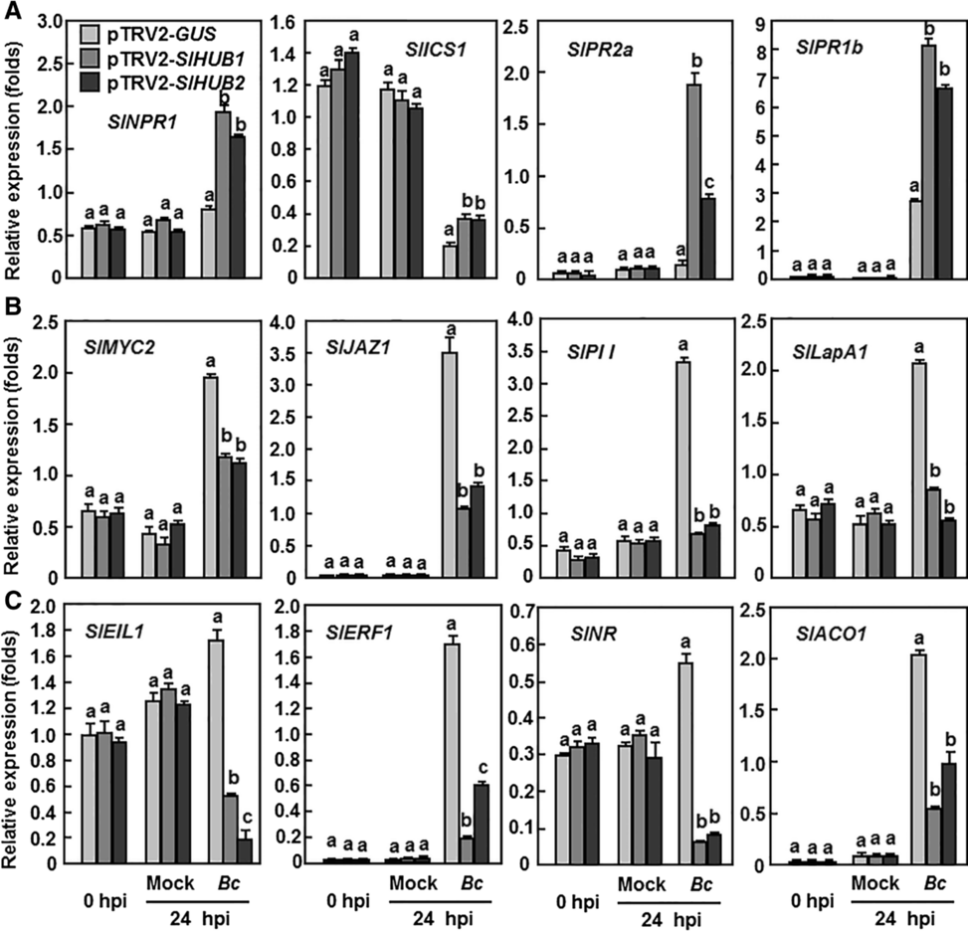
Silencing of *SlHUB1* and *SlHUB2* affected the expression of SA-, JA- and ET-mediated signaling and responsive genes after *Botrytis* infection. Two-week-old seedlings were infiltrated with agrobacteria carrying pTRV2-*SlHUB1*, pTRV2-*SlHUB2*, pTRV2-*SlMED21* or pTRV2-*GUS* construct and were inoculated with spore suspension of *B. cinerea* at 4 weeks after VIGS infiltration. At least 6 leaves from 6 individual plants were collected at 0 and 24 h after inoculation and used for analysis of gene expression. **a** Expression of SA-mediated signaling and responsive genes, (**b**) Expression of JA-mediated signaling and responsive genes and (**c**) Expression of ET-mediated signaling and responsive genes. Relative expression levels were shown as folds of the actin transcript values. Data presented in (**b**) are the means ± SD from three independent experiments and different letters above the columns indicate significant differences at *p* < 0.05 level

To explore the signaling pathways that were associated with the function of SlHUB1 and SlHUB2 in the disease resistance to *B. cinerea*, we further analyzed and compared the expression changes of the JA/ET- and SA-mediated signaling genes and their corresponding defense-related genes in the pTRV2-*SlHUB1*-, pTRV2-*SlHUB2*- and pTRV2-*GUS*-infiltrated plants before and after infection of *B. cinerea*. Four genes, e.g. *SlNPR1*, *SlICS1*, *SlPR1b* and *SlPR2b*, that are associated with SA biosynthesis and signaling or regulated by the SA-mediated signaling [64, 65]; four genes, e.g. *SlMYC2*, *SlJAZ1*, *SlPII* and *SlLapA1*, that are involved in or regulated by the JA-mediated signaling [66–68], and four genes, e.g. *SlEIL1*, *SlERF1*, *SlNR* and *SlACO1*, that are involved in or regulated by the ET-mediated signaling [69, 70], were chosen. In the healthy (0 h after inoculation) and mock-inoculated plants, the expression levels of the tested genes in the pTRV2-*SlHUB1*- and pTRV2-*SlHUB2*-infiltrated plants were similar to but the expression levels of *SlICS1* was higher than those in the pTRV2-*GUS*-infiltrated plants (Fig. 9). After inoculation with *B. cinerea*, the expression levels of *SlNPR1* and *SlPR2a* were increased slightly but the expression level of *SlPR1b* was dramatically increased by >100-fold and *SlICS1* was significantly decreased by ~5-fold in the pTRV2-*GUS*-infiltrated plants, as compared with those in the mock-inoculated plants (Fig. 9a). However, the expression levels of the four SA-mediated signaling genes *SlNPR1*, *SlICS1*, *SlPR2a* and *SlPR1b* in the pTRV2-*SlHUB1*- and pTRV2-*SlHUB2*-infiltrated plants were significantly increased as compared with those in the pTRV2-*GUS*-infiltrated plants (Fig. 9a). Notably, the expression level of *SlICS1* in the pTRV2-*SlHUB1*- and pTRV2-*SlHUB2*-infiltrated plants was also decreased by ~3-fold after inoculation with *B. cinerea* compared with it in the mock-inoculated plants but still significantly higher than it in the pTRV2-*GUS*-infiltrated plants. By contrast, the expression of the JA-mediated signaling-related genes *SlMYC2*, *SlJAZ1*, *SlPII* and *SlLapA1* and the ET-mediated signaling-related genes *SlEIL1*, *SlERF1*, *SlNR* and *SlACO1* in the pTRV2-*GUS*-infiltrated plants were drastically induced upon infection of *B. cinerea*, as compared with those in the mock-inoculated plants (Fig. 9b and c). However, the expression levels of all these genes in the pTRV2-*SlHUB1*-, pTRV2-*SlHUB2*-infiltrated plants were markedly decreased, showing 0.5 ~ 10-fold of reduction, as compared with those in the pTRV2-*GUS*-infiltrated control plants, at 24 h after inoculation with *B. cinerea* (Fig. 9b and c). These results indicate that silencing of either *SlHUB1* or *SlHUB2* significantly attenuates the JA/ET-mediated signaling and defense response but selectively activated the SA-mediated signaling and defense response upon infection of *B. cinerea*.

## Discussion

### Differential requirement and different functions of SlHUB1 and SlHUB2 in disease resistance against *B. cinerea* and *Pst* DC3000

Recent studies have demonstrated that the Arabidopsis histone H2B monoubiquitination E3 ligases AtHUB1 and AtHUB2 play critical roles in regulating growth and development [36, 37, 39–44] as well as in modulating immune response against pathogens and pathogen-derived toxin [13, 45, 46]. In the present study, we characterized the tomato orthologues of the Arabidopsis AtHUB1/AtHUB2, SlHUB1 and SlHUB2 (Fig. 1a). Both of SlHUB1 and SlHUB2 exhibited histone H2B monoubiquitination E3 ligases activity *in vitro* (Fig. 1c) and their expression could be induced by pathogens such as *B. cinerea* and *Pst* DC3000 and by defense-signaling related hormones (Fig. 2). In Arabidopsis, it was recently demonstrated that the Arabidopsis AtHUB1 is required for disease resistance against *B. cinerea* and *Alternaria brassicicola*, another necrotrophic fungal pathogen, whereas the function of AtHUB2 in disease resistance remains unclear [13]. We found in the present study that silencing of either *SlHUB1* or *SlHUB2* resulted in increased disease severity and *in planta* fungal growth (Fig. 3). In our study, sequences of the VIGS fragments for *SlHUB1* and *SlHUB2* were quite divergent at nucleotide level and the transcript levels of *SlHUB1* and *SlHUB2* in the *SlHUB2*- and *SlHUB1*-silenced plants were similar to the control plants (Fig. 3a), indicating that the increased disease phenotype observed in the *SlHUB1*- and *SlHUB2*-silenced plants is not caused by a simultaneous co-silencing of both *SlHUB1* and *SlHUB2*. Therefore, it is likely that both *SlHUB1* and *SlHUB2* are required for resistance to *B. cinerea* in tomato. On the other hand, unlike the Arabidopsis *AtHUB1* and *AtHUB2* that do not have function in resistance to *Pst* DC3000 and the obligate fungal pathogen *Erysiphe cichoracearum*, the causal agent of powdery [13, 45], silencing of *SlHUB1* led to a reduced severity of disease caused by *Pst* DC3000 (Fig. 5), indicating that at least SlHUB1 plays a role in resistance to *Pst* DC3000. This is further supported by the observation that the SA-mediated signaling, which is considered to regulate disease resistance to *Pst* DC3000, could be activated in the *SlHUB1*- or *SlHUB2*-silenced plants upon infection of *B. cinerea* (Fig. 9a). Evidence presented in this study suggests that the tomato SlHUB1 and SlHUB2 positively regulate resistance against *B. cinerea* while only SlHUB1 negatively regulate resistance against *Pst* DC3000. In addition, the Arabidopsis AtHUB1 and AtHUB2 were found to regulate the expression of some *R* genes such as *SUPPRESSOR OF npr1-1, CONSTITUTIVE1* (*SNC1*) and *RESISTANCE TO PERONOSPORA PARASITICA4*, indicating an impact of AtHUB1 and AtHUB2 on immune responses in Arabidopsis [45]. Collectively, it is likely that the plant HUB1/HUB2 and the HUB1/HUB2-mediated H2B monoubiquitination play differential roles in disease resistance against pathogens.

It was recently reported that the Arabidopsis AtHUB1 and AtHUB2 can form both homodimers and heterodimers *in vivo* [42] and does not have overlapping function in regulating the expression of *SNC1* [45]. This nature might partially explain the differential requirements of *SlHUB1* and *SlHUB2* in disease resistance to *B. cinerea* and *Pst* D3000, that is, formation of heterodimers or homodimers of SlHUB1 and SlHUB2 in response to different stimuli or signals from invading pathogens may play different roles in resistance against pathogens. Another, the Arabidopsis AtMED21, a subunit of an evolutionarily conserved Mediator complex that is thought to play a key role in regulating RNA polymerase II activity [71], was found to interact with AtHUB1 and play critical roles in disease resistance to necrotrophic fungi and embryo development [13]. In the present study, we found that the tomato SlMED21 did interact with SlHUB1 but not with SlHUB2 (Fig. 4a) and that silencing of *SlMED21* did not affect the phenotypes of diseases caused by *B. cinerea* and *Pst* DC3000 (Figs. 4 and 5). Thus, it is likely that SlMED21 does not function in resistance against *B. cinerea* and *Pst* DC3000, providing a distinct mechanism for action of SlHUB1 in disease resistance from that of the Arabidopsis AtHUB1 [13]. However, whether SlHUB1 interacts with other subunits of the Mediator complex is worthy to be further examined because the interaction with Mediator complex was proposed as an important mode required for functions of AtHUB1 in disease resistance [13].

### SlHUB1 and SlHUB2 regulate multiple defense responses in tomato plants upon infection of *B. cinerea*

During infection process, necrotrophic fungi like *B. cinerea* often secrete a series of cell-wall degrading enzymes to destroy the cell wall barrier of plant cells and cause leakage of nutrients that can be extracted by the invading pathogen for growth and reproduction [72–74]. In this regard, the integrity and strength of the cell wall in plants are thought to play an important role in resistance to *B. cinerea* [72]. It was found that inoculation of tomato leaves with *B. cinerea* induced a reinforcement of the cell wall at the site of fungal entry [75]. In the present study, we found that the cell wall thickness of the *SlHUB1*- or *SlHUB2*-silenced plants was markedly reduced compared with the control plants (Fig. 6a and b). Similar observation was also obtained in the Arabidopsis *athub1* mutant plant, which showed reduced cell wall thickness [13]. Most importantly, upon infection of *B. cinerea*, the pathogen-induced expression of genes involved in the phenylpropanoid pathway (e.g. *SlPALs* and *SlC4H*) and cell wall formation (e.g. *SlCADs*) in the *SlHUB1*- or *SlHUB2*-silenced plants were significantly suppressed (Fig. 7), indicating that silencing of *SlHU1* and *SlHUB2* may lead to defects in the effective responsiveness of these cell wall-related genes and thus in cell wall formation during pathogenic infection. Another, it was also found that the cuticle layer in leaves of the Arabidopsis *athub1* and *athub2* mutant plants was irregularly disorganized and the expression of some genes involved in cutin and wax biosynthesis was downregulated in the *athub1* and *athub2* mutants [38]. Given that the cell wall formation is closely linked to phenylpropanoid pathway [58], which was responsible for lignification to strengthen the cell wall [76], we thus conclude that defects in cell wall in the *SlHUB1*- or *SlHUB2*-silenced plants may account for, at least partially, the reduced resistance to *B. cinerea*. In addition to cell wall biosynthesis, *PAL* genes are also important for SA biosynthesis [77]. However, the majority of pathogen-induced SA production occurs via a distinct pathway, isochorismate synthase (ICS1) [77]. Whether *PAL* genes here are involved in SA biosynthesis need to be investigated further.

It is well documented that ROS play important roles in the establishment of infection by some necrotrophic pathogens such as *B. cinerea* [72]. Previous works showed that *B. cinerea* can utilize ROS for establishment of infection [78–80], although it was also reported that resistance to *B. cinerea* in *sitiens*, an ABA-deficient tomato mutant, involves timely production of H_2_O_2_ [75]. In this study, we observed that significant accumulation of H_2_O_2_ and superoxide anion in the *SlHUB1*- or *SlHUB2*-silenced plants after infection of *B. cinerea*, although the accumulation of H_2_O_2_ and superoxide anion in the *SlHUB1*- or *SlHUB2*-silenced plants without infection had no obvious difference with the control (Fig. 7a and b), indicating that silencing of *SlHUB1* or *SlHUB2* may loss the control of ROS generation and scavenging upon pathogen infection. This hypothesis is supported by the expression changes of the genes involved in ROS generation and scavenging in the *SlHUB1*- or *SlHUB2*-silenced plants. For example, the expression levels of *SlRboh1*, which can reduce the accumulation of H_2_O_2_ when silenced [45, 63], were significantly increased while the expression levels of *SlSOD1* and SlGR1, which are involved in ROS scavenging, were decreased in the *SlHUB1*- or *SlHUB2*-silenced plants after infection of *B. cinerea* (Fig. 7c). The upregulated expression of *SlCAT1* and *SlAPX5* in the *SlHUB1*- or *SlHUB2*-silenced plants after infection of *B. cinerea* might be due to their feedback regulation by the excess accumulation of ROS in the cells (Fig. 7a and b). It seems likely that silencing of *SlHUB1* or *SlHUB2* promotes the *B. cinerea*-induced accumulation of ROS through a perturbation on the expression of genes in ROS generation and scavenging and thereby attenuates disease resistance to this pathogen.

Consistent with previous observation in tomato-*B. cinerea* interaction [75, 81], we found in this study that infection of *B. cinerea* induced significant accumulation of callose at the infection sites (Fig. 6c and d). We also found in the present study that silencing of *SlHUB1* or *SlHUB2* led to increased accumulation of callose at the infection sites in the *SlHUB1*- or *SlHUB2*-silenced plants after infection of *B. cinerea* (Fig. 6c and d). This is consistent with the observation that the Arabidopsis *athub1* mutant plants accumulated increased levels of callose upon infection of *B. cinerea* [13]. However, the role of callose accumulation in disease resistance seems complicated [73]. Whereas reduced amounts of callose accumulation was found to be associated with increased susceptibility to *A. brassicicola*, loss of callose had no effect on resistance to *B. cinerea* [13]. It seems that SlHUB1 and SlHUB2, together with the Arabidopsis AtHUB1 [13], may have functions in regulating accumulation of callose at the infection site; however, it is unlikely that these accumulated callose contributes to resistance against *B. cinerea*. This is contrast to previous observations supporting a role of callose accumulation as a part of defense response to *B. cinerea* in tomato [75, 82].

### SlHUB1 and SlHUB2 contribute to tomato resistance against *B. cinerea* through balancing the SA- and JA/ET-mediated pathways

SA, JA, and ET all independently contribute in different ways to resistance of tomato to *B. cinerea*. The involvement of the Arabidopsis AtHUB1 and AtHUB2 in disease resistance was already documented [13, 42]. AtHUB1 acts independently of JA but ET and SA are involved in modulating the resistance of athub1 mutants to necrotrophic fungi [13]. However, the signaling pathway that AtHUB1 and AtHUB2 might be involved is largely unknown yet. In the present study, the expression of genes involved in the SA-, JA- and ET-mediated signaling pathways exhibited different patterns in the *SlHUB1*- and *SlHUB2*-silenced plants upon infection of *B. cinerea* (Fig. 9), providing new insights into the possible SlHUB1- and SlHUB2-regulated signaling pathway. SA is a defense molecule that modulates plant resistance to diverse pathogens but increased SA was shown to be associated with susceptibility to necrotrophic fungal pathogens including *B. cinerea* [61]. In this study, the expression of *SlICS1*, encoding an isochorismate synthase involved in the synthesis of SA [81], was suppressed by *B. cinerea* in the control plants (Fig. 9a), indicating that the tomato plants can suppress the biosynthesis of SA and thus reduce the endogenous SA level to defend the infection of *B cinerea*. However, in the SlHUB1- and SlHUB2-silenced plants, the expression of the SA-mediated signaling regulatory genes *SlICS1* and *SlNPR1* and defense genes *SlPR2b* and *SlPR1b* was significantly upregulated after infection of *B cinerea* (Fig. 9a), implying a boosted SA-mediated signaling, which may attenuate the defense response *to B. cinerea*. This is partially supported by several recent observations that *B. cinerea* can manipulate and use the SA-mediated signaling pathway to promote disease development in tomato [83, 84] and that SA-promoted disease development occurs through NPR1, which can be induced by *B. cinerea* [83]. On the other hand, it is generally believed that resistance to *B. cinerea* requires both the JA- and ET-mediated signaling pathways in Arabidopsis [85, 86]. In tomato, activation of the JA/ET-dependent defense pathway, is also required for resistance to *B. cinerea* [83, 87]. In the present study, the expression of the JA- and ET-mediated signaling and responsive genes was markedly induced by *B. cinerea* in control plants (Fig. 9b), indicating that active JA- and ET-mediated signaling pathways could be initiated upon infection of *B. cinerea*. By contrast, the expression of these JA- and ET-mediated signaling and responsive genes in the *SlHUB1*- and *SlHUB2*-silenced plants was suppressed significantly after infection of *B. cinerea* (Fig. 9b and c). This implies that silencing either *SlHUB1* or *SlHUB2* resulted in attenuated JA- and ET-mediated signaling and thereby decreased defense response, which led to the reduced resistance to *B. cinerea*, demonstrating the importance of both the JA- and ET-mediated signaling pathways in SlHUB1- and SlHUB2-regulated resistance to *B. cinerea*. However, it is not clear that whether the JA- and ET-mediated signaling pathways act independently or in combination in the functions of SlHUB1 and SlHUB2. JA was found to act independently of ET in inducing resistance to *B. cinerea* in tomato [87]. Furthermore, like those in Arabidopsis, antagonistic cross-talks among the SA-, JA-and ET-mediated signaling pathways in tomato resistance to *B. cinerea* were also reported [83]. Collectively, our data support that SlHUB1 and SlHUB2 exert their functions in resistance to *B. cinerea* through modulating the balance between the JA/ET- and SA-mediated signaling pathways.

## Conclusion

In sum, we present evidence supporting that both SlHUB1 and SlHUB2 contribute to resistance to *B. cinerea* in tomato through modulating the balance between the SA- and JA/ET-mediated pathways. Further studies, e.g., profiling of gene expression between the *SlHUB1*- or *SlHUB2*-silenced plants and non-silenced plants after infection of *B. cinerea* and analysis of histone H2B monoubiquitination at specific gene loci, will be helpful to the mechanism of SlHUB1 and SlHUB2 in tomato resistance to *B. cinerea*.

## Availability of supporting data

Phylogenetic data was deposited in the LabArchives under the DOI ‘10.6070/H49G5JTB’ (https://mynotebook.labarchives.com/share/Dayong%2520Li/MjIuMXwxMDIwMDkvMTcvVHJlZU5vZGUvNDAwMzM4NDQ2NHw1Ni4x).

## Additional files

Additional file 1:
**Primers used in this study for different purposes.** (PDF 50 kb) Additional file 2:
**Silencing efficiency in pTRV2-** ***SlPDS*** **-infiltrated tomato plants. Two-week-old seedlings were infiltrated with agrobacteria carrying pTRV2-** ***SlPDS*** **or pTRV2-** ***GUS*** **and leaf samples were collected 3 weeks after VIGS treatment.** (A) Phenotype of pTRV2-*SlPDS*- or pTRV2-*GUS*-infiltrated tomato plants. (B) The transcript of *SlPDS* was analysed by qRT-PCR. (PDF 3931 kb) Additional file 3:
**SlMED21 is an orthologue of AtMED21.** (A) Comparisons of tomato SlMED21 protein with Arabidopsis AtMED21 protein. (B) Neighbor-joining tree of SlMED21 from tomato and other eukaryotic organisms. The MED21 amino acid sequences from various organisms were used for building the phylogenetic tree. Sequence alignment was carried out by the ClustalX program. Phylogenetic trees were constructed using the neighbor-joining (NJ) method of the MEGA6 program with the p-distance and complete deletion option parameters. The reliability of the obtained trees was tested using a bootstrapping method with 1000 replicates. The MED21 sequences are from *Sorgum bicolor* (SbMED21, AAL73528), *Oryza Sative* (OsMED21, BAD03057), Arabidopsis (AtMED21, AAD03443, At4g04780), *Solanum lycopersicum* (SlMED21, AK328398), *Mus Musculus* (MmMED21, AAH12286), *H. sapiens* (HsMED21, NP_004255), *Danio rerio* (DrMED21, NP_998588), *S. cervisae* (ScMED21, P47822), *Schizosaccharomyces pombe* (SpMED21, CAA22343), *Physcomitrella patens* (PpMED21, XP_001763794) and *Anopheles gambiae* (AgMED21, XP_307937). The AtMED21 and SlMED21 were marked in red. (PDF 3007 kb)
