# Supplementary material for: Tomato histone H2B monoubiquitination enzymes SlHUB1 and SlHUB2 contribute to disease resistance against Botrytis cinerea through modulating the balance between SA- and JA/ET-mediated signaling pathways
Source: BMC Plant Biol. 2015 Oct 21;15:252. doi: 10.1186/s12870-015-0614-2 (PMC4618151; doi:10.1186/s12870-015-0614-2)
Supplement: Additional file 1: — Primers used in this study for different purposes. (PDF 50 kb) [file 12870_2015_614_MOESM1_ESM.pdf]

Additional file 3: Primers used in this study for different purposes.

Primers	Sequences (5’-3’)	Size (bp)
Cloning		
SlHUB1-race- 1R	GCTTATCTCTCGCCTTATCGT	350
SlHUB1-race- 2R	TAAGTGTGGATTCCATATCCT	
SlHUB1-race- 3R	GCTATTCTGGAATCAGCAATGGT	
SlHUB2-race- 1R	GACTCAGCTTCATTAGCAGCT	1700
SlHUB2-race- 2R	AGCAGCAGAAAGCCTCTGCTGA	
SlHUB2-race- 3R	TGACCAATCGTCTCAATCTCAGA	
SlHUB1-1F	ATG GAAAACTTGCTACCGCTTG	2544
SlHUB1-1R	TCAGATATAAACGGCCTTGACA	
SlHUB2-1F	ATGGAGAGCTCGGCTGCTGCAT	2652
SlHUB2-1R	TCAGATTTTCACAAAACGAATG	
SlMED21-1F	ATGGATATCATATCACAGTTAC	417
SlMED21-1R	TCATTCTGGCTTCTTCAAGTTC	
VIGS		
SlMED21-VIGS-1F	GCGTCTAGAATGGATATCATATCACAGTTAC	319
SlMED21-VIGS-1R	ATACTCGAGGAAGTTCTTGGCCAACTGCAT	
SlHUB1-VIGS-1F	GCGGAATTCTGAAGCTCAGAAGATTGAGA	443
SlHUB1-VIGS-1R	ATACTCGAGACTTTTGGAGGCATGATCCATC	
SlHUB2-VIGS-1F	GCGGAATTCAGCTAGATGATGATCTGATTA	554
SlHUB2-VIGS-1R	ATACTCGAGGCTGACTGCCTTTGATTACAA	
SlPDS-VIGS-1F	CGGTCTAGAGGCACTCAACTTTATAAACC	409
SlPDS-VIGS-1R	CGGGGATCCCTTCAGTTTTCTGTCAAACC	
GUS-VIGS-1F	CGGTCTAGAACCTGGGTGGACGATATCAC	396
GUS-VIGS-1R	CGGGGATCCGTGCACCATCAGCACGTTAT	
qRT-PCR		
SlMED21-rt-1F	GATTTGGAAGATGGATATCA	150
SlMED21-rt-1R	GTGGGATTAGCAGGTGGAGG	
SlHUB1-rt-1F	CAACTGTAGATAACGTTAGGCA	196
SlHUB1-rt-1R	GTATCTCTATGATTCTGCAATG	
SlHUB2-rt-1F	CTTATGATGACTTCTTGGTTAC	182
SlHUB2-rt-1R	CAACACCCTACACAGGAATATC	
SlNPR1-rt-1F	TGTGGGAAAGATAGCAGCACG	147

SlNPR1-rt-1R	GTCCACACAAACACACACATC	
SlPII-rt-1F	GTTGTACAAATGCCTGTGGTGAC	135
SlPII-rt-1R	GGTAAGAGTACATGAAGAGATGC	
SlLAPA1-rt-1F	CATAGCAACTGGAGTTGTGC	153
SlLAPA1-rt-1R	CCTGCACATACATGTTCTGC	
SlPAL1-rt-1F	AGGCTCAATCTGTGTTCTTTCC	168
SlPAL1-rt-1R	CATGTCATCATGTTCACAAAGC	
SlPAL2-rt-1F	AGCAATGTGCAATGGACAGATC	149
SlPAL2-rt-1R	AATTGAATGAACATACAATTTAGAG	
SlPAL4-rt-1F	GTTGTGAATGCTTGTCTAGTGC	184
SlPAL4-rt-1R	ATAGACATAAGCACACTGTCAC	
SlPAL6-rt-1F	TGAGACAGTGCAAGCGACATAG	138
SlPAL6-rt-1R	GTCTTCTTGAAAGAAGCCACAA	
SlC4H-rt-1F	GCCAATTCTTGGCATCACCATT	194
SlC4H-rt-1R	CATCACAATGGTGGAATGCTTC	
SlPAL3-rt-1F	GTTAGAATGCCTTAAGGAATGG	198
SlPAL3-rt-1R	GCTACAAACCAATATATTCAAGAG	
SlPAL5-rt-1F	GGCCATATGTAATATTGTCTATC	135
SlPAL5-rt-1R	TTGTCGTAGTGGGCGTGATTAA	
SlSOD-rt-1F	GGCCAATCTTTGACCCTTTATG	183
SlSOD-rt-1R	AAGTCCAGGAGCAAGTCCAGTT	
SlCAT1-rt-1F	CCCAGTTAATGCTCCCAAGTGT	118
SlCAT1-rt-1R	AGGACGACAAGGATCAAACCTC	
SlAPX5-rt-1F	ACTTCACGGAGCTTTTGAGTGG	141
SlAPX5-rt-1R	CAGCATAGTCAGCAAAGAAGGC	
SlGR1-rt-1F	GATGATGAAATGCGAGCTGTAG	182
SlGR1-rt-1R	TTTGTGTTAGGGAGACGACCAG	
SlRBOH1-rt-1F	GTTGCTGCAGCCATTGTCAC	130
SlRBOH1-rt-1R	GGCTTGGGCCAAAATCATTC	
SlICS1-rt-1F	TCGCCGGCATTCATTGGAAACA	183
SlICS1-rt-1R	GCACTCCCGTACTATAGCAAAC	
SlPR1b-rt-1F	CCGTGCAATTGTGGGTGTC	106
SlPR1b-rt-1R	GAGTTGCGCCAGACTACTTG	
SlPR2b-rt-1F	GGCAGGAACACCAAAGAAACCA	127
SlPR2b-rt-1R	TGGCCTCTGGTCAGGTTTAAAG	

SlMYC2-rt-1F	TAGCCACACTGGAGGCAAGATT	140
SlMYC2-rt-1R	CTAGGTCTAATTCCATGAGCGC	
SlJAZ2-rt-1F	GGAAACCTGATCAACCAGAG	125
SlJAZ2-rt-1R	GGGGTTCTGTTTGTTGGCTA	
SlNR-rt-1F	TGCGGTTATGGTTCTGGTTCTC	142
SlNR-rt-1R	ATGGGCTCGCATGGAATCTTCT	
SlERF1-rt-1F	TGGAGTTAGAAAGAGGCCATGG	143
SlERF1-rt-1R	CCCTCATTGATAATGCGGCTTG	
SlACO1-rt-1F	CATGTCCTAAGCCCGATTTG	107
SlACO1-rt-1R	TTTGAGGAGTTGAAGGCCAC	
SlEIL1-rt-1F	CTGAAGATGGGCAAAGGATG	127
SlEIL1-rt-1R	ACACGAGGTTGTTGATGAGG	
SlActin-1F	AGGCACACAGGTGTTATGGT	177
SlActin-1R	AGCAACTCGAAGCTCATTGT	
BcActin-1F	TCCAAGCGTGGTATTCTTACCC	117
BcActin-1R	TGGTGCTACACGAAGTTCGTTG	
SlPDS-rt-1F	GCCAAAAGAAGTTGTCGGAAGC	129
SlPDS-rt-1R	ACTTGCTTCACCTCGCACTCTT	
Monoubiquitination activity		
SlHUB1-e-1F	ATAGCGGCCGCATGGAAAACTTGCTACCGCTT	2544
SlHUB1-e-1R	ATACTCGAGTCAGATATAAACGGCCTTGACA	
SlHUB2-e-1F	ATAGCGGCCGCATGAAAGAATTAAGGGACAAA	2652
SlHUB2-e-1R	ATACTCGAGTCAGATTTTCACAAAACGAATG	
SlHUB1-eRING-1F	ATAGCGGCCGCATGGAAAACTTGCTACCGCTT	2385
SlHUB1-eRING-1R	ATACTCGAGTCAATTGAGTATTTCCTTGTATTC	
SlHUB2-eRING-1F	ATAGCGGCCGCATGAAAGAATTAAGGGACAAA	2490
SlHUB2-eRING-1R	ATACTCGAGTCAGAGAATAGCCTTGGAATCAT	
Yeast two-hybrid assay		
SlHUB1-AD-1F	GCTGTACAAGGGATCCATGGAAAACTTGCTACCGCTTG	2544
SlHUB1-AD-1R	TAATTAACTCTCTAGATCAGATATAAACGGCCTTGACA	
SlMED21-BD-1F	CGCGGATCCATGGATATCATATCACAGTTAC	417
SlMED21-BD-1R	TGCTCTAGATCATTCTGGCTTCTTCAAGTTC	
