# Supplementary figures and images for: Tomato histone H2B monoubiquitination enzymes SlHUB1 and SlHUB2 contribute to disease resistance against Botrytis cinerea through modulating the balance between SA- and JA/ET-mediated signaling pathways
Source: BMC Plant Biol. 2015 Oct 21;15:252. doi: 10.1186/s12870-015-0614-2 (PMC4618151; doi:10.1186/s12870-015-0614-2)

**(A)**

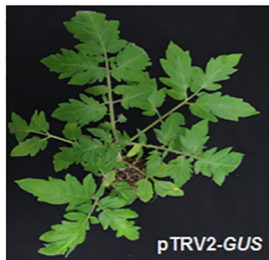

Total plants                      13  
Bleaching plants                0

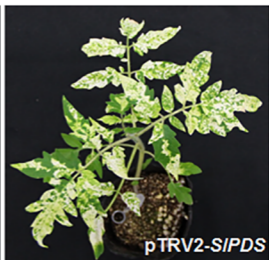

13  
11

**(B)**

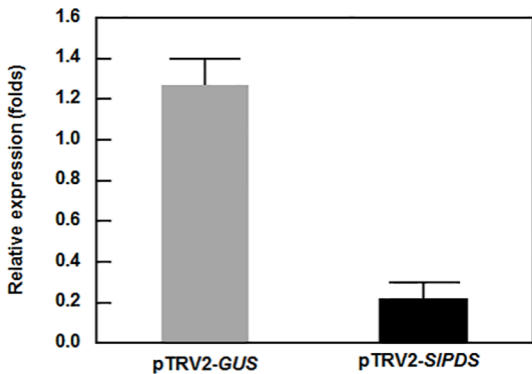

Supplement: Additional file 2: — Silencing efficiency in pTRV2- SlPDS -infiltrated tomato plants. Two-week-old seedlings were infiltrated with agrobacteria carrying pTRV2- SlPDS or pTRV2- GUS and leaf samples were collected 3 weeks after VIGS treatment. (A) Phenotype of pTRV2-SlPDS- or pTRV2-GUS-infiltrated tomato plants. (B) The transcript of SlPDS was analysed by qRT-PCR. (PDF 3931 kb) [file 12870_2015_614_MOESM2_ESM.pdf]
