# Supplementary material for: Tomato histone H2B monoubiquitination enzymes SlHUB1 and SlHUB2 contribute to disease resistance against Botrytis cinerea through modulating the balance between SA- and JA/ET-mediated signaling pathways
Source: BMC Plant Biol. 2015 Oct 21;15:252. doi: 10.1186/s12870-015-0614-2 (PMC4618151; doi:10.1186/s12870-015-0614-2)
Supplement: Additional file 3: — SlMED21 is an orthologue of AtMED21. (A) Comparisons of tomato SlMED21 protein with Arabidopsis AtMED21 protein. (B) Neighbor-joining tree of SlMED21 from tomato and other eukaryotic organisms. The MED21 amino acid sequences from various organisms were used for building the phylogenetic tree. Sequence alignment was carried out by the ClustalX program. Phylogenetic trees were constructed using the neighbor-joining (NJ) method of the MEGA6 program with the p-distance and complete deletion option parameters. The reliability of the obtained trees was tested using a bootstrapping method with 1000 replicates. The MED21 sequences are from Sorgum bicolor (SbMED21, AAL73528), Oryza Sative (OsMED21, BAD03057), Arabidopsis (AtMED21, AAD03443, At4g04780), Solanum lycopersicum (SlMED21, AK328398), Mus Musculus (MmMED21, AAH12286), H. sapiens (HsMED21, NP_004255), Danio rerio (DrMED21, NP_998588), S. cervisae (ScMED21, P47822), Schizosaccharomyces pombe (SpMED21, CAA22343), Physcomitrella patens (PpMED21, XP_001763794) and Anopheles gambiae (AgMED21, XP_307937). The AtMED21 and SlMED21 were marked in red. (PDF 3007 kb) [file 12870_2015_614_MOESM3_ESM.pdf]

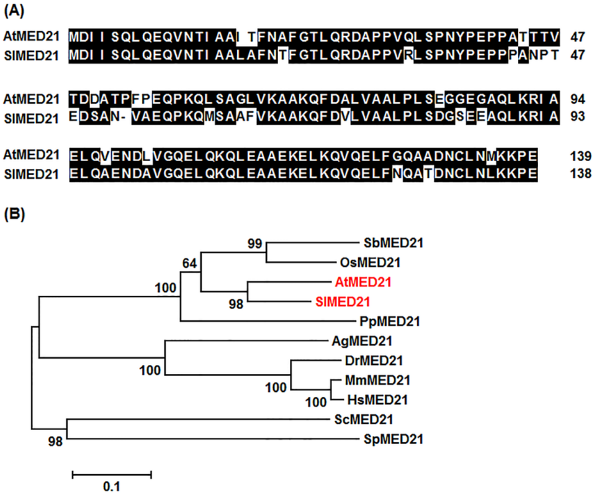
(A)
AtMED21 MDIISQLQEQVNTIAAITFNAFGTLQRDAPPVQLSPNYPEPPATTTV 47
SlMED21 MDIISQLQEQVNTIAALAFNTFGTLQRDAPPVRLSPNYPEPPPANPT 47
AtMED21 TDDATPFPEQPKQLSAGLVKAAKQFDALVAALPLSEGGEGAQLKRIA 94
SlMED21 EDSAN-VAEQPKQMSAAFVKAAKQFDVLVAALPLSDGSEEAQLKRIA 93
AtMED21 ELQVENDLVGQELQKQLEAAEKELKQVQELFGQAADNCLNMKKPE 139
SlMED21 ELQAENDAVGQELQKQLEAAEKELKQVQELFNQATDNCLNLKKPE 138
(B)
SbMED21
OsMED21
AtMED21
SlMED21
PpMED21
AgMED21
DrMED21
MmMED21
HsMED21
ScMED21
SpMED21
99
64
100
98
100
100
100
98
0.1
